# A systematic review of how homeopathy is represented in conventional and CAM peer reviewed journals

**DOI:** 10.1186/1472-6882-5-12

**Published:** 2005-06-14

**Authors:** Timothy Caulfield, Suzanne DeBow

**Affiliations:** 1 Health Law Institute, University of Alberta, Canada

## Abstract

**Background:**

Growing popularity of complementary and alternative medicine (CAM) in the public sector is reflected in the scientific community by an increased number of research articles assessing its therapeutic effects. Some suggest that publication biases occur in mainstream medicine, and may also occur in CAM. Homeopathy is one of the most widespread and most controversial forms of CAM. The purpose of this study was to compare the representation of homeopathic clinical trials published in traditional science and CAM journals.

**Methods:**

Literature searches were performed using Medline (PubMed), AMED and Embase computer databases. Search terms included "homeo-pathy, -path, and -pathic" and "clinical" and "trial". All articles published in English over the past 10 years were included. Our search yielded 251 articles overall, of which 46 systematically examined the efficacy of homeopathic treatment. We categorized the overall results of each paper as having either "positive" or "negative" outcomes depending upon the reported effects of homeopathy. We also examined and compared 15 meta-analyses and review articles on homeopathy to ensure our collection of clinical trials was reasonably comprehensive. These articles were found by inserting the term "review" instead of "clinical" and "trial".

**Results:**

Forty-six peer-reviewed articles published in a total of 23 different journals were compared (26 in CAM journals and 20 in conventional journals). Of those in conventional journals, 69% reported negative findings compared to only 30% in CAM journals. Very few articles were found to be presented in a "negative" tone, and most were presented using "neutral" or unbiased language.

**Conclusion:**

A considerable difference exists between the number of clinical trials showing positive results published in CAM journals compared with traditional journals. We found only 30% of those articles published in CAM journals presented negative findings, whereas over twice that amount were published in traditional journals. These results suggest a publication bias against homeopathy exists in mainstream journals. Conversely, the same type of publication bias does not appear to exist between review and meta-analysis articles published in the two types of journals.

## Background

Growing popularity of complementary and alternative medicine (CAM) in the public sector is reflected in the scientific community by an increased number of research articles assessing efficacy and therapeutic effects. At the same time, there is increasing concern about how research results are disseminated and communicated to both professionals and the public (AMA) [1]. Indeed, there is some suggestion that publication bias occurs in both mainstream medicine and with CAM [2]. Given the impact that the scientific literature has on the perception of treatments, particularly within the conventional health care community, it is worthwhile to gain an understanding of how various forms of scientific investigation are represented in the relevant literature. This seems particularly so in the context of CAM, where there has been some criticism about how the conventional scientific community has addressed many CAM treatments [3]. As such, in this systematic review, we seek to explore the difference between how "conventional" and "CAM" peer reviewed journals represent CAM research.

Homeopathy is one of the most widespread and most controversial forms of CAM. A reasonable amount of clinical research has been done on homeopathy (though the quality of the research varies substantially) and the efficacy of many homeopathic remedies remains unclear [4,5]. Moreover, despite its relative popularity among the general public, it has (rightly or not) struggled to gain legitimacy in the medical establishment – particularly in North America [6]. Because homeopathy is a treatment that is likely to evoke a spectrum of responses (ranging from acceptance to deep skepticism), it seems an ideal CAM treatment to study in this context. Indeed, it is an area of study where balance is particularly important. As noted in one paper: "An unbiased conclusion is of utmost importance in this domain because it is a scientific, emotional and political issue in many areas of the world." [7].

## Methods

Relevant homeopathic papers reporting on clinical trials were collected. Literature searches were performed using Medline (PubMed), AMED and Embase computer databases. Search terms included "homeo-pathy, -path, and -pathic" and "clinical" and "trial." All articles published in English over the past 10 years were included (1994 through to August 2004). Our search yielded 251 articles overall, of which 46 systematically examined the efficacy of homeopathic treatment. An examination of 15 meta-analyses on homeopathic efficacy was also performed. Review and meta-analysis articles were found by inserting the term "review" instead of "clinical" and "trial". These review articles, all of which analyzed and commented on homeopathic clinical trials, were also used to ensure that our collection of studies was reasonably comprehensive.

If the study or review was published in a journal that specialized in CAM generally (e.g., *The Journal of Alternative and Complementary Medicine*) or homeopathy (e.g., *Homeopathy *and the *British Homeopathic Journal*) it was classified as appearing in a "CAM journal." If the study or review appeared in a general medical journal (e.g., *Lancet*) or a non-CAM specialty journal (e.g., *European Journal of Clinical Pharmacology*), it was classified as appearing in a "conventional journal." We categorized the general conclusions of the study (positive or negative results). In addition, we did an analysis of the general tone of the articles. If the article used language that seemed explicitly negative against homeopathy (e.g., condemnation of the general area), it would be categorized as a "negative tone." If the piece used language that was optimistic or encouraging of the area, it was categorized as "positive."

## Results

### Clinical trials

We examined 46 peer-reviewed articles published in a total of 23 different journals. Twenty-six experiments published in conventional journals were compared with twenty articles published in CAM journals. Of those in conventional journals, 69% (18/26) reported negative findings compared with only 30% (6/20) of CAM journals reporting negative findings.

In our analysis of the general tone of the articles, we found only a few that were clearly negative. However, those that were negative appeared in conventional journals and used relatively harsh language. For example, in one study it was stated "homeopathy is pure quackery" [8]. That said, most of the research papers presented the results in a relatively balanced fashion. We categorized 10 of the 26 conventional journal articles and 8 of the 20 CAM journal articles as having a positive tone. The rest were deemed to be "neutral." Many of the papers categorized as neutral suggested possible clinical benefit despite a negative result. For example, one study published in a conventional journal suggested that " [a]lthough the overall results of the study were negative, they do not rule out the possibility that individual patients may benefit from this homeopathic treatment" [9].

### Review articles

A total of the 15 meta-analyses/systematic reviews were examined (10 conventional, 5 CAM). The most common conclusions in the reviews are that the existing evidence remains inconclusive and that more high quality research is required. The conclusions of the reviews were ambiguous enough to make it difficult to categorize them as either a strictly "positive" or "negative" finding. Nevertheless, we can say that none of these reviews had an overtly negative tone (e.g., none of the papers completely dismissed homeopathy, even in the face of negative conclusions). Six articles in the conventional journals and two in the CAM journals had a neutral tone. The rest of the review articles were somewhat positive, encouraging further research and/or suggesting possible clinical benefit. For example, some of the systematic review articles were encouraging of the area, despite skepticism about the possible biological mechanism underlying homeopathy. This occurred in both the conventional and CAM journals. Indeed, we felt that most of the review articles sought to present a balanced picture of the available evidence associated with homeopathy. That said, there were a number of notable differences between the papers published in the conventional and CAM journals.

Almost all of the systematic reviews in conventional journals start on a skeptical note. Indeed, 9 out of 10 of the articles begin with a statement that questions the scientific plausibility of homeopathy. Some of the articles use relatively strong language to make the point. For example, Ernst and Pittler suggest that it is the use of "highly diluted material that overtly flies in the face of science and has caused homeopathy to be regarded as placebo therapy at best and quackery at worst" [10]. Others merely raise the point that there are scientific issues associated with homeopathy and then provide an opposing perspective. McCarney et al., summarize the debate as follows: "The molecules contained in a homeopathic remedy are diluted beyond Avogadro's number. This has led some investigators to question whether homeopathic therapy could have any effect over placebo. However, proponents of homeopathy claim that the remedies act through biophysical pathways, and all include the idea of some form of information transfer from the diluted substance to the diluting agent" [11].

Of the systematic reviews published in CAM journals, only one mentions the issue of "scientific implausibility" and goes on to suggest that "homeopathy's possible mechanisms of action remain intangible theories, and it will be important ultimately to substantiate these" [12]. The other four review articles published in CAM journals begin with defensive statements, noting the benefits of homeopathy (e.g., low side effects) and the challenges of using conventional clinical trial and systematic review methods to assess homeopathic treatments [13].

Despite these differences in approach, all of the systematic reviews are relatively evenhanded, noting arguments on either side of the debates surrounding efficacy and research methodology.

## Discussion

Some scholars have speculated about a possible trend toward publishing negative results in conventional journals. For example, Cucherat, et al., have suggested "homeopathy trials with 'negative' results might be more readily accepted by non-homeopathic journals, since the lack of efficacy of homeopathy is in accordance with the belief of many non-homeopathic physicians." [5]. The results of this study provide some preliminary evidence to support this claim. While a small study with clear limitations, there was a stark difference between the numbers of studies that were negative in the conventional journals (69%) as compared to the CAM journals (30%).

That said, publication bias – that is, a journal favoring the publication of positive or negative results – is only one possible explanation of this apparent trend. There may also be a submission bias. For instance, are studies with a negative result submitted to conventional journals and those with a positive to CAM journals? Without access to submission patterns, it will be difficult to analyze this issue. However, it is worth noting that an examination of the affiliations of the first authors of the clinical trials revealed that there was no clear pattern regarding where medical doctors and homeopathic experts publish. In other words, the apparent discipline (and this was not always clear) and home institution of the first author was not a predictor of where an article is published.

Our analysis of the systematic reviews also has some interesting implications. Though there is some evidence of a possible bias in the publication of clinical trials (toward the negative in the conventional journals and toward the positive in the CAM journals), there does not appear to be a similar trend with reviews. In addition, these articles seemed relatively balanced in the presentation of results. Given that there is often significant opportunity to editorialize in systematic reviews, this conclusion demonstrates that the authors (and the editors of journals) are striving to explore this controversial area in a relatively impartial manner. This conclusion does not necessarily conflict with our data that suggests a publication bias. A publication bias would likely be an inadvertent systemic problem (i.e., not an explicit policy) whereas the tone of the articles would reflect apparent writing and editorial decisions.

## Conclusion

This small study has a number of clear limitations. For example, we only considered articles that were published in English and we did not critique the quality of the study (might higher quality studies be published in more well known conventional journals?). In addition, aside from the data on whether the conclusions were positive or negative, much of the analysis was subjective. Nevertheless, is does provide some preliminary evidence of a possible publication bias and insight on the general tone of publications involving homeopathy.

## Competing interests

The author(s) declare that they have no competing interests.

## Authors' contributions

Both Caulfield and Debow contributed to the design of the study, the analysis of the data and the writing and editing of the manuscript.

## Pre-publication history

The pre-publication history for this paper can be accessed here:


