# A systematic review of how homeopathy is represented in conventional and CAM peer reviewed journals

**DOI:** 10.1186/1472-6882-8-31

**Published:** 2008-06-17

**Authors:** Timothy Caulfield, Suzanne DeBow

**Affiliations:** 1 Health Law Institute, University of Alberta, Canada

It has come to our attention that there are several referencing errors in the published version of our article [1]. The errors are small and do not impact the conclusions of the paper. Nevertheless, we feel it is important to recognize and correct the mistakes, particularly since some involve quotes.

In the second paragraph of the Results section, the first quote should include only the word "quackery" and not the entire phrase. Similarly, the last quote should only include that phrase after the comma (i.e., "they do not rule out the possibility that individual patients may benefit from this homeopathic treatment"). On page 3 in the Discussion section's 1st paragraph, we incorrectly cite reference 5 for a quote that is out of reference 7. For reference 6, the correct page numbers are 92–111. And for reference 3 the page numbers are 1–10.

## Pre-publication history

The pre-publication history for this paper can be accessed here:



## References

[B1] Caulfield T, Debow S (2005). A systematic review of how homeopathy is represented in conventional and CAM peer reviewed journals. BMC Complementary and Alternative Medicine.

